# Medullary thyroid carcinoma – Adverse events during systemic treatment: risk-benefit ratio

**DOI:** 10.1590/2359-3997000000267

**Published:** 2017-06-14

**Authors:** Léa Maria Zanini Maciel, Patrícia Künzle Ribeiro Magalhães

**Affiliations:** 1 Departamento de Medicina Interna Faculdade de Medicina de Ribeirão Preto Universidade de São Paulo Ribeirão Preto SP Brasil Divisão de Endocrinologia, Departamento de Medicina Interna, Faculdade de Medicina de Ribeirão Preto da Universidade de São Paulo (FMRP-USP), Ribeirão Preto, SP, Brasil

**Keywords:** Medullary thyroid carcinoma, systemic treatment, tyrosine kinase inhibitors, adverse effects

## Abstract

Medullary thyroid carcinoma (MTC) is a rare neuroendocrine tumor originating from parafollicular C cells of the thyroid and associated with mutations in the proto-oncogene **RE**arranged during **T**ransfection (RET). The prognosis of MTC depends on clinical stage, with a 95.6% 10-year survival rate among patients with localized disease and 40% among patients with advanced disease. Standard chemotherapy and radiotherapy have no significant impact on the overall survival of these patients and two tyrosine kinase receptor inhibitors (TKIs), vandetanib and cabozantinib, have been recently approved for the systemic treatment of locally advanced or metastatic MTC. However, since patients with MTC and residual or recurrent disease may have an indolent course with no need for systemic treatment, and since these drugs are highly toxic, it is extremely important to select the patients who will receive these drugs in a correct manner. It is also essential to carefully monitor patients using TKI regarding possible adverse effects, which should be properly managed when occurring.

## INTRODUCTION

Medullary thyroid carcinoma (MTC) represents 1-2% of all thyroid carcinomas in the United States, a lower frequency than previously reported (3-5%), primarily due to the higher relative incidence of papillary thyroid cancer (PTC) over the last 3 decades (1). MTC is a rare neuroendocrine tumor originating from parafollicular C cells of the thyroid and associated with mutations in the proto-oncogene **RE**arranged during **T**ransfection (*RET*) (1,2).

MTC is associated with activators of the *RET* proto-oncogene, which codes for a membrane receptor of the tyrosine-kinase type of 150-170 kD highly expressed in normal and tumor cells derived from the neural crest, including parafollicular thyroid cells. The RET receptor, after interacting with one of its ligands and undergoing dimerization, activates the RAS-RAF-MAP kinase and PI3K-AKT pathways, being responsible for the transduction of extracellular signals of cell proliferation, growth, differentiation and survival, and cell apoptosis. Genomic mutations activating the *RET* proto-oncogene are present in most patients with hereditary MTC, with the existence of a genotype-phenotype correlation. In addition to the *RET*, the superexpression of receptors 2 and 3 of the vascular endothelial growth factor (VEGF), which leads to tumor angiogenesis and nutrition, and of the epidermal growth factor receptor (EGFR), involved in tumor proliferation, also contributes to the pathogenesis of MTC (3).

MTC occurs in a sporadic or non-hereditary manner in 75-80% of the patients. In the remaining cases (20-25%), it is an autosomal dominant hereditary disease with a high degree of penetrance and variability of expression, which may be part of two distinct clinical syndromes depending on the organs involved, *i.e.,* type 2A multiple endocrine neoplasia (MEN2A) or type 2B multiple endocrine neoplasia (MEN2B). MEN2A, which accounts for 95% of MEN2 cases, can be divided into four variants: classical MEN2A (represented by the uniform presence of MTC and the less frequent occurrence of pheochromocytoma or hyperparathyroidism, or both), MEN2A with cutaneous lichen amyloidosis, MEN2A with Hirschsprung’s disease, and familial MTC (families or individuals with *RET* germline mutations who have MTC but not pheochromocytoma or hyperparathyroidism). Patients with MEN2B develop MTC and pheochromocytoma and exhibit a recognizable phenotype, characterized by a typical facies, ophthalmologic abnormalities (inability to make tears in infancy, thickened and everted eyelids, mild ptosis, and prominent corneal nerves), skeletal malformations (marfanoid body habitus, narrow long facies, pes cavus, pectus excavatum, high-arched palate, scoliosis, and slipped capital femoral epiphyses), and a generalized ganglioneuromatosis throughout the aerodigestive tract (1).

MTC has a variable survival depending on the clinical stage. Ten-year survival is 95.6% among patients with localized disease, 75.5% among patients with extension of the disease to nearby tissues or presence of metastatic ganglia, and 40% among patients with distant metastases (4). However, distant metastases are detected in 7-17% of the patients at diagnosis (5). Nevertheless, a substantial number of patients with distant metastases may have an indolent disease course or a slow progression for several years.

The main pathway of MTC dissemination is the lymphatic one, with a high frequency of macroscopic or microscopic metastases to regional lymph nodes. The incidence of metastases correlates with the size of the primary tumor, with metastases being present in more than 75% of palpable tumors (6). Other common sites of metastasis are the mediastinum, hilar ganglia, lungs, liver, and bones and, less frequently, skin and brain (7).

These tumors secrete calcitonin (Ct) and carcinoembryonic antigen (CEA), which are sensitive biomarkers of the disease. The postoperative Ct and CEA values and their doubling times (DT) are predictors of aggressive tumor behavior. Patients with a Ct-DT of more than 1 year have a 95% 10-year survival rate and a 73% 5-year recurrence-free survival rate. Conversely, patients with Ct-DT of 1 year or less have a 10-year survival rate of 18% and a survival rate without disease recurrence of 20% (8).

The only treatment potentially inducing a cure of MTC is surgery, with the most important initial treatment being the full removal of the tumor and suspected ganglia in order to prevent relapses. In about 50% of cases, the disease is unresectable at the time of diagnosis (9). When cervical metastases are detected on the occasion of the initial surgery, the cure rate is low and residual disease will be revealed radiologically or biochemically in 90% of the patients (10).

Patients with persistent cervical disease can be placed under observation or submitted to a new surgery if progression is detected. Many patients with distant metastases have indolent disease and do not require systemic therapy for many years. Localized therapy with external irradiation can be considered as a palliative treatment for painful bone metastases or for the prevention of other events, such as compression of the spinal cord or fracture. Other measures can also be taken for progressive and symptomatic bone metastatic disease such as radiotherapy and/or antiresorptive medications (intravenous bisphosphonates or RANK ligand inhibitors). Embolization or cryoablation of metastatic liver or bone disease may be beneficial in some cases, relieving pain and treating refractory diarrhea (11).

Only a selected group of patients with metastatic MTC should be considered for systemic therapy. These are patients with significant disease progression over a period of fewer than 12-14 months, with symptomatic tumor growth or involvement of vital organs precluding the possibility of localized therapy.

### Systemic treatment

Knowledge about the pathways involved in MTC tumorigenesis has led to the testing of various tyrosine kinase receptor inhibitors (TKI) for the treatment of this tumor. Vandetanib was the first of them to be approved for the treatment of progressive, locally advanced, and unresectable or metastatic MTC in April 2011 in the United States, in February 2012 in Europe, and in November 2012 in Brazil. Cabozantinib was the other drug approved in the United States in November 2012 and in Europe in March 2014 for the treatment of progressive, locally advanced, and unresectable or metastatic MTC. This medication has not yet been approved by Anvisa and is not available in Brazil for the treatment of MTC.

### Vandetanib

Vandetanib is a potent TKI of the RET receptor and of receptors 2 and 3 of VEGF (VEGF-2 and VEGF-3) and also has inhibitory activity on EGFR (12,13). Its approval was based on a phase III, randomized, placebo-controlled, double-blind study (ZETA) which demonstrated a longer progression-free survival (PFS) estimated at 30 months in patients with MTC treated with vandetanib compared with the placebo group (PFS = 19 months; *p* < 0.001) (14). The overall response to vandetanib was 44%. A demonstration of progression before patient admission to the study was not a criterion for entry, a fact that may have contributed to the PFS detected in the placebo group. Additionally, crossover of the placebo group to the group treated with vandetanib was permitted upon demonstration of tumor progression, an event that may also have been a confounding factor in the PFS analysis.

Although TKIs are better tolerated than cytotoxic chemotherapy, many patients experience side effects. In the ZETA study, treatment with vandetanib led to a low discontinuation rate due to toxicity (12%).

The main toxic effects of any grade are listed in Table 1. The adverse effects most commonly detected are diarrhea, skin rash, acne, fatigue, nausea, arterial hypertension, headache, vomiting, prolonged QT in the electrocardiogram (ECG), and photosensitivity. The most common laboratory abnormalities are hypocalcemia, increased levels of hepatic enzymes, hypoglycemia, hypothyroidism, and increased creatinine levels. Rare but serious adverse events may occur, including Stevens-Johnson syndrome, interstitial pulmonary disease, ischemic stroke, congestive heart failure, and posterior leukoencephalopathy syndrome (15,16). Only 2.5% of the patients treated with vandetanib have been reported to suffer a fatal adverse effect.

**Table 1:** Toxicity of any degree related to the use of vandetanib

Toxicity	Laboratory abnormalities
Diarrhea, 57%	Reduction of calcium, 57%
Rash, 53%	Increase of aspartate transaminase, 51%
Acneiform dermatitis/acne, 35%	Reduction of glycemia, 24%
Nausea, 33%	Increase of alkaline phosphatase, 53%
Hypertension, 33%	Increase of creatinine, 16%
Headache, 26%	Increase of bilirubins, 13%
Fatigue, 24%	Reduction of magnesium, 7%
Reduced appetite, 21%	Reduction of potassium, 6%
Abdominal pain, 21%	Increase of potassium, 6%
Dry skin, 15%	Reduction of sodium, 6%
Vomiting, 15%	Neutropenia, 10%
QTc prolongation*, 14%	Thrombocytopenia, 9%
Photosensitivity reaction, 13%	
Pruritus, 11%	
Dyspepsia, 11%	
Proteinuria, 10%	
Depression, 10%	

QTc: corrected QT interval. * Prolonged QTc > 450 ms occurred in 65% and > 500 ms in 7% using Fridericia’s correction.

Adapted from Cabanillas and cols. (17).

A reduction of the dose from 300 to 200 mg/day is recommended in the presence of more advanced adverse effects (grade 3 or 4) of any nature. A new reduction of the dose to 100 mg/day should be prescribed if an additional grade 3 or 4 adverse effect should occur (17,18).

Before starting treatment, possibly present electrolyte disorders such as hypocalcemia, hypokalemia, and hypomagnesemia should be corrected, and a baseline ECG should be obtained. These tests should be repeated 2 to 4 weeks and 8 to 12 weeks after the beginning of treatment and then every 3 months for proper monitoring. Throughout treatment, the patient should be instructed not to take any medication without the approval of the physician since some medications can prolong the QT interval. Patients with a history of long QT syndrome or uncorrectable electrolyte disorder should not be treated with this medication.

Recently, Fox and cols. (19) conducted a phase I/II trial for adolescents (13-18 years) and children (5-12 years) with metastatic or locally advanced MTC. Sixteen patients were treated with vandetanib 100 mg/m^2^/day. The authors concluded that this dosage is well tolerated and highly active for adolescents and children with locally advanced or metastatic MTC and MEN2B.

### Cabozantinib

Cabozantinib is a multikinase inhibitor of VEGFR2, RET, and hepatocyte growth factor receptor (c-MET), which was approved for the treatment of MTC after the demonstration of PFS advantages in the group treated with the TKI compared with the placebo group (11.2 months versus 4 months, respectively, *p* < 0.0001) in a phase III, randomized, double-blind study conducted on 330 patients with MTC and radiologically confirmed disease progression (20). The recommended dose is 140 mg/day. However, because of the high toxicity of the drug, lower initial doses have been suggested (100 or 120 mg/day) (21), with later increases to the full dose if the patients tolerate it or if the desired response is not achieved. The drug should be ingested on an empty stomach, and perforations, fistulas, and hemorrhages may occur. The drug should be used with care or avoided in patients with a history of cervical irradiation or disorders of the gastrointestinal mucosa. The side effects more commonly detected with the use of this drug are listed in Table 2.

**Table 2:** Toxicity of any degree related to the use of cabozantinib

Toxicity	Laboratory abnormalities
Diarrhea, 63%	Increase of alanine transaminase, 86%
Stomatitis, 51%	Increase of aspartate transaminase, 86%
Hand-foot syndrome, 50%	Increase of alkaline phosphatase, 53%
Weight loss, 48%	Reduction of calcium, 52%
Reduced appetite, 46%	Reduction of phosphorus, 28%
Nausea, 43%	Increase of bilirubins, 25%
Fatigue, 41%	Reduction of magnesium, 19%
Dysgeusia, 34%	Reduction of potassium, 18%
Changes in hair color, 34%	Reduction of sodium, 10%
Hypertension, 33%	Neutropenia, 35%
Constipation, 27%	Thrombocytopenia, 35%
Abdominal pain, 27%	Increase of TSH, 57%
Vomiting, 24%	
Dysphonia, 20%	
Headache, 18%	
Dysphagia, 13%	
Dyspepsia, 11%	

Adapted from Cabanillas and cols. (17).

### The choice of a TKI

The choice of which of the approved TKIs should be used for the beginning of treatment is unclear. The first study discussing this aspect was published by Cabanillas and cols. in 2014 (17). The idea is that this choice should be centered on the patient, taking into consideration his clinical history, the changes detected upon physical examination, and the results of baseline laboratory tests. Since vandetanib is known to possibly lead to prolongation of the QT interval, “torsade de pointes*”*, and sudden death, the drug should not be administered to patients with long QT syndrome, arrhythmias, decompensated heart failure, or uncorrectable electrolyte disorders. Also, the drug should not be administered to patients with a corrected QT (QTc) of more than 450 milliseconds and should be prescribed with caution to patients taking an antiarrhythmic drug.

Cabozantinib can cause perforation, fistulas, and hemorrhages. When medical history is taken, the patient should be questioned about the presence of hemoptysis and hemorrhages, diverticulitis, chronic gastrointestinal disease (Crohn’s disease and ulcerative colitis), active peptic ulcer, or radiation treatment of the neck or mediastinum, since these patients will have a higher risk of gastrointestinal perforation, hemorrhage, and tracheoesophageal fistulas. Invasion of the trachea, bronchi, and esophagus increases the risk of formation of a fistula. Thus, in these situations, vandetanib will be better indicated.

Also, the use of vandetanib is preferable for patients whose profession requires the frequent use of the hands (*e.g.*, musicians) since the hand-foot syndrome is a frequent side effect of cabozantinib. In addition, vandetanib is associated with weight gain and cabozantinib with weight loss (22).

A review of the drugs used by the patient is important. The concomitant use of CYP3A4 inhibitors may increase the toxicity of cabozantinib by increasing its plasma concentrations. In contrast, the use of CYP3A4 inducers can reduce the plasma concentrations of vandetanib and, consequently, the efficacy of the drug (23,24).

## CONCLUSIONS

TKIs represent a highly valuable therapy for patients with metastatic and/or locally advanced MTC. However, it is extremely important to establish an appropriate stratification of the clinical risk of the patient to whom these drugs will be administered, since a significant number of patients with MTC and residual or recurrent disease have an indolent course characterized by stable or slowly increasing Ct and/or CEA levels. The prescription of TKI is not indicated for these patients in view of the toxicity of these drugs, the lack of their curative power, and the need for their chronic use when they are prescribed.

In addition, careful monitoring of the patients using these drugs and the management of side effects that might arise with treatment are important for the safe maintenance of the patient and for the determination of the time when the drug loses its efficacy.

Disclosure: Léa Maria Zanini Maciel is a principal investigator of AstraZeneca studies (ZETA, KALYPSO, VERIFY and ASTRA). Patrícia Künzle Ribeiro Magalhães is a subinvestigator of AstraZeneca studies (ZETA, KALYPSO, VERIFY and ASTRA).
